# Patients with Crohn’s disease achieving transmural healing experience superior long-term outcomes compared with those with endoscopic or radiologic healing alone

**DOI:** 10.1093/ibd/izag114

**Published:** 2026-07-03

**Authors:** Sudheer Kumar Vuyyuru, Shane W Goodwin, Virginia Solitano, Darryl Ramsewak, Zahra Kassam, Cassandra Townsend, Jamie Gregor, Melanie Beaton, Eileen Crowley, Maan Alkhattabi, Michael Sey, Vipul Jairath

**Affiliations:** Division of Gastroenterology, Department of Medicine, Western University, London, ON, Canada; Lawson Health Research Institute, Western University, London, ON, Canada; Division of Gastroenterology and Gastrointestinal Endoscopy, IRCCS Ospedale San Raffaele, Università Vita-Salute San Raffaele, Milan, Italy; Division of Epidemiology and Biostatistics, Western University, London, ON, Canada; Department of Medical Imaging, Western University, London, ON, Canada; Department of Medical Imaging, Western University, London, ON, Canada; Division of Gastroenterology, Department of Medicine, Western University, London, ON, Canada; Division of Gastroenterology, Department of Medicine, Western University, London, ON, Canada; Division of Gastroenterology, Department of Medicine, Western University, London, ON, Canada; Division of Pediatric Gastroenterology, Department of Pediatrics, Children’s Hospital Western Ontario, Western University, London, ON, Canada; Department of Medicine, Faculty of Medicine, King Abdulaziz University, Rabigh, Jeddah, Saudi Arabia; Division of Gastroenterology, Department of Medicine, Western University, London, ON, Canada; Lawson Health Research Institute, Western University, London, ON, Canada; Division of Gastroenterology, Department of Medicine, Western University, London, ON, Canada; Lawson Health Research Institute, Western University, London, ON, Canada; Division of Epidemiology and Biostatistics, Western University, London, ON, Canada

**Keywords:** Crohn’s disease, transmural healing, remission, relapse

## Abstract

**Background:**

Crohn’s disease (CD) is characterized by transmural inflammation, and achieving transmural healing (TH), may lead to improved long-term outcomes. However, evidence supporting this hypothesis is limited.

**Methods:**

Adult patients diagnosed with CD who underwent ileocolonoscopy and cross-sectional imaging within 6 months were included. Long-term outcomes of CD-related surgery and hospitalization in patients with TH, endoscopic healing (EH) alone, radiologic healing (RH) alone, and no healing (NH) were assessed. We used Cox proportional hazards models and modified Poisson regression.

**Results:**

Among 180 included patients, 26.7% achieved TH, 21.1% achieved EH, 12.8% achieved RH, and 39.4% were classified as NH. At baseline, 54.4% were on biologics which increased to 81.7% at last follow-up (76-78 months across groups). Cumulative probabilities of surgery were lowest in patients with TH (0%, 2.3%, 7.0% at 1, 3, and 5 years) and highest in patients with NH (13%, 20.5%, and 26.9%, respectively) (*P* = .03). On Cox proportional hazard regression analysis, TH was associated with a significantly reduced risk of CD-related surgery (hazard ratio, 0.01; 95% confidence interval, 0.00-0.35; *P* = .009) compared with NH. The probabilities of surgery were lower for patients with RH compared with EH (10% vs 13.2%, 10% vs 18.4%, and 21.2% vs 26.9% at 1, 3, and 5 years) but were not statistically significant (*P* = .41). Probabilities of CD-related hospitalization were lowest with TH (4.3%, 8.8%, and 13.6% at 1, 3 and 5 years) but were not statistically different than other healing categories (*P* = .67).

**Conclusions:**

TH was associated with superior long-term outcomes compared with EH or RH alone. Future studies should focus on standardizing its definition and evaluate treat-to-target strategies.

Key Messages **What is already known?** 

Emerging evidence suggests that transmural healing may be associated with better long-term outcomes. However, there is insufficient evidence to support this claim.

**What is new here?** 

We found that patients achieving transmural healing experience significantly lower risk of long-term Crohn’s disease–related surgery and hospitalization.

**How can this study help patient care?** 

Our study offers valuable insights into the long-term risk of surgery and hospitalization across different healing types and contributes to the limited existing evidence in this area.

## Introduction

Crohn’s disease (CD) is characterized by transmural inflammation^1^ and often leads to stricturing or penetrating complications requiring bowel resection.^2^ Disease activity in patients with CD can be assessed through various modalities, including clinical symptoms, biomarkers, endoscopy, and cross-sectional imaging. Traditionally, symptom resolution has been the cornerstone treatment target in the management of CD; however, approximately 50% of patients with CD in clinical remission may still exhibit signs of mucosal inflammation on colonoscopy,^3^ Furthermore, achieving endoscopic healing (EH) has been associated with improved long-term outcomes.^4^ Consequently, international consensus guidelines, such as STRIDE-II (Selecting Therapeutic Targets in Inflammatory Bowel Disease–II),^5^ recommend EH as a long-term treatment target. Nonetheless, endoscopic assessment reflects inflammation limited to the intestinal mucosa and may not accurately represent the full burden and extent of intestinal inflammation in CD, given the transmural nature of the disease.

Cross-sectional imaging techniques such as computed tomography (CT), magnetic resonance imaging (MRI), and intestinal ultrasound (IUS) can assess transmural inflammation.^6^^,^^7^ Moreover, patients with CD who demonstrate EH in the terminal ileum may still have active inflammation more proximally, which is beyond the reach of ileocolonoscopy.^8^ A recent systematic review reported that achievement of transmural healing (TH) is associated with a higher likelihood of clinical remission and lower rates of intestinal resection.^9^ However, this evidence is based on a limited number of observational studies. Therefore, to address the gaps in the current literature, we evaluated the long-term outcomes in patients with CD who achieve EH, radiologic healing (RH), and TH.

## Methods

### Study population and design

This is a retrospective cohort study that included adult patients (≥18 years of age) with a confirmed and established diagnosis of CD based on standard clinical, endoscopic, and histologic criteria. Eligible participants were patients with CD who underwent ileocolonoscopy with terminal ileal intubation as well as cross-sectional imaging (computed tomography enterography [CTE] or magnetic resonance enterography [MRE]) within the 3-month period from January 1, 2010, to December 31, 2018, at London Health Sciences Center and St. Joseph’s Health Center in London, Canada. These patients underwent imaging studies and ileocolonoscopy as part of evaluation of disease activity during the treatment course of inflammatory bowel disease (IBD). Patients with a prior proctocolectomy with an end ileostomy or ileal pouch–anal anastomosis were excluded. The reporting of this study follows STROBE (Strengthening the Reporting of Observational Studies in Epidemiology) guidelines.

### Definitions of healing

EH was defined as the absence of ulceration including aphthous ulcers on ileocolonoscopy but not meeting the definition of RH.^10^ RH was defined as no sign of active inflammation (ie, thickened bowel wall, ulceration, edema, diffusion-restriction, or increased contrast enhancement), no extraenteric sign (ie, fat creeping, enlarged lymph nodes, or comb sign), and no CD-related complications (ie, stricture, fistula, or abscess) on cross-sectional imaging but not meeting the definition of EH.^11^ TH was defined as meeting the definition of both EH and RH.^11^ No healing (NH) was defined as presence of inflammation on both ileocolonoscopy and cross-sectional imaging.

### Image acquisition

CTE and MRE were performed using local standard protocols. Patients ingested 500 to 750 mL of an iso-osmotic lactulose–water solution as a negative oral contrast over 45 to 60 minutes. For MRE, an additional 250 mL of water or Metamucil was administered 15 minutes before imaging. CTE was acquired in the enteric phase, 45 seconds after intravenous administration of 100 mL of iodinated contrast (Omnipaque 350; GE Healthcare). Multiplanar reconstructions with high spatial resolution (slice thickness ≤3 mm) were generated in axial, coronal, and sagittal planes. MRE was performed on a 1.5-T MRI scanner using an 8-channel phased-array body coil, with gadolinium contrast and an antispasmodic agent. High-resolution ultrafast sequences (true fast imaging with steady-state precession) were obtained prior to intravenous injection of 20 mg hyoscine butylbromide. Following hyoscine butylbromide, axial and coronal single-shot fast spin-echo T2-weighted images, coronal fat-saturated T2-weighted images, and diffusion-weighted imaging images were acquired. Coronal and axial fat-suppressed 3-dimensional T1-weighted breath-hold gradient-echo images of the abdomen and pelvis were obtained before and after intravenous gadolinium-based contrast (Gadovist; Bayer) administration with dynamic imaging across 4 phases for the coronal sequences.

### Image evaluation

Radiology reports of patients with IBD who underwent CT and MRI during the time period were identified, and these were reviewed for the presence or absence of radiologic features suggestive of small bowel inflammation, strictures, or fistulas. The radiologic criteria used to define small bowel inflammation included 1 or more of the following components: (1) mural hyperenhancement, (2) mural thickening, (3) mural/mucosal ulceration(s), (4) transmural edema, (5) mesenteric fat inflammation (increased fat attenuation or T2 signal intensity), and (6) the comb sign (hyperemia and engorgement of the vasa recta). Skip lesions were defined as the discontinuous pattern of inflammation along the gastrointestinal tract identified on cross-sectional imaging.^11^

### Ethical considerations

Ethical approval for medical chart review was granted by the Western University Research Ethics Board through a delegated and full board review, providing waiver of need for patient consent.

### Statistical analysis

The key outcomes assessed were time to CD-related surgery and CD-related hospitalization. Potential risk factors associated with risk of CD-related surgery (bowel resection because of CD-related indications) and CD-related hospitalization were evaluated, and cumulative probabilities for both outcomes were calculated, along with log-rank test to examine for statistical differences in the cumulative probabilities across time and among the healing groups. Group comparisons based on healing category were performed using chi-square tests or Fisher exact tests for categorical variables and 1-way analysis of variance or Kruskal-Wallis test for continuous variables, as appropriate. To explore differences in the time to long-term CD-related outcomes across healing groups and the risk factors associated with each, we utilized Cox proportional hazards models. To report differences in relative risk of outcomes across healing groups, we utilized modified Poisson regression, treating each outcome as a binary indicator of event occurrence by end-of-cohort follow-up.^12^ To assess potential confounders, we assessed the association of outcomes to baseline characteristics (sex, smoking status, age, disease duration, biologic use, prior surgery status, C-reactive protein [CRP], hemoglobin concentration, albumin concentration, white blood cell concentration, inflammation proximal to terminal ileum, and presence of strictures on endoscopy). Characteristics found to be significant at an alpha level of 0.20 in univariate models were kept in the final multivariable model, following literature recommendations^9^^,^^10^ and supplemented with review by clinical expertise to ensure that important characteristics were not missed. In all multivariable models, statistical significance was defined as a *P* value <.05. To examine effect measure modification, an interaction term between type of healing and CRP was included in the models. Significant interactions indicate that there is a difference in effects between those with nonhigh vs high levels of CRP. All analyses were conducted using R version 4.4.1 (R Foundation for Statistical Computing).^11^^,^^13^

## Results

### Baseline characteristics

A total of 180 patients were included in the final analysis (Figure S1). The baseline and imaging characteristics of participants by type of healing are shown in Tables 1 and 2. Among the included patients, 40.5% (n = 73) were male and mean disease duration was 12.5  ± 12.1 years. Approximately half of the included patients (54.4% [n = 98]) were on advanced therapies at baseline, and at the end of follow-up 81.7% (n = 147 of 180) were on advanced therapies. A history of prior bowel resection for CD was present in 39.4% (n = 71). Among the cross-sectional imaging studies, 41% (n = 74) were CTE and the remaining were MRE. The median duration between imaging study and ileocolonoscopy was 1 month (interquartile range: 2 months, Q1-Q3: 0-2 months).

**Table 1:** Baseline characteristics of included patients.

Parameter	Type of healing				
	**No healing (n** = **71)**	**Endoscopic healing (n** = **38)**	**Radiological healing (n** = **23)**	**Transmural healing (n** = **48)**	*P* value
**Female**	59.2 (42)	68.4 (26)	43.5 (10)	60.4 (29)	.29
**Age, y**	44.9 ± 16.5	42.4 ± 18.0	40.0 ± 14.6	46.0 ± 15.4	.44
**Smoking status**					.86
** Never or prior smoker**	83.1 (59)	79.0 (30)	87.0 (20)	85.4 (41)	
** Current smoker**	16.9 (12)	21.1 (8)	13.0 (3)	14.6 (7)	
**Disease duration, y**	12.4 ± 12.8	9.9 ± 9.6	10.5 ± 10.9	15.6 ± 13.0	.14
**Advanced therapies at baseline**	46.5 (33)	65.8 (25)	56.5 (13)	56.3 (27)	.27
**Perianal surgery**	11.3 (8)	0.0 (0)	8.7 (2)	16.7 (8)	.04
**Previous resection**	40.9 (29)	26.3 (10)	43.5 (10)	45.8 (22)	.29
**Type of imaging study**					.07
**CTE**	46.5 (33)	29.0 (11)	26.1 (6)	50.0 (24)	
**MRE**	53.5 (38)	71.0 (27)	73.9 (17)	50.0 (24)	
**CRP >5 mg/L**	39.4 (28)	26.3 (10)	39.1 (9)	16.7 (8)	.02
**Hb, g/L**	132.0 ± 17.9	132.0 ± 14.7	135.0 ± 15.1	136.0 ± 19.2	.38
**Albumin, g/L**	40.6 ± 4.3	41.2 ± 4.1	41.8 ± 4.5	43.3 ± 5.0	.01
**Duration between imaging study and ileoscolonoscopy, mo**	1 [2]	1 [1.75]	1 [2]	1 [2]	.48
**Duration of follow-up, mo**	76 [15]	76.5 [18.5]	77 [43.5]	78 [14.75]	.91

Values are % (n), mean ± SD, or median [interquartile range].

Abbreviations: CRP, C-reactive protein; CTE, computed tomography enterography; Hb, hemoglobin; MRE, magnetic resonance enterography.

**Table 2.** Features of inflammation on cross-sectional imaging.

Parameter	Type of healing				
	No healing (n = 71)	Endoscopic healing (n = 38)	Radiological healing (n = 23)	Transmural healing (n = 48)	*P* value
**Mural ulceration**	2.8 (2)	2.6 (1)	0.0 (0)	0.0 (0)	.74
**Mural hyperenhancement**	59.2 (42)	63.2 (24)	0.0 (0)	0.0 (0)	<.001
**Mural thickening**	83.1 (59)	79.0 (30)	0.0 (0)	0.0 (0)	<.001
**Intramural edema**	0.0 (0)	2.6 (1)	0.0 (0)	0.0 (0)	.34
**Mesenteric fat inflammation**	8.5 (6)	5.3 (2)	0.0 (0)	0.0 (0)	.12
**Comb sign**	1.4 (1)	0.0 (0)	0.0 (0)	0.0 (0)	1.00
**Penetrating complications**	5.6 (4)	5.3 (2)	0.0 (0)	0.0 (0)	.28

Values are % (n).

### Types of healing

Of included patients, 26.7% (n = 48) met the criteria of TH, whereas EH was noted in 21% (n = 38), RH in 12.8% (n = 23), and NH in 39.4% (n = 71). Median follow-up ranged from 76 to 78 months across groups. There were some differences in baseline characteristics among the groups (Table 1). Patients achieving TH were more likely to have had a perianal surgery (16.7%) compared with 11.3%, 0.0%, and 8.7% in the in NH, EH, and RH groups, respectively (*P* = .04) and were more less likely to have a CRP >5  mg/L (16.7% in TH vs 39.4% in NH, 26.3% in EH, and 39.1% in RH; *P* = .02). A greater proportion of patients with EH were on advanced therapies (65.8% [n = 25]) at baseline compared with other groups (TH: 56.3%; RH: 56.5%; NH: 46.5%), and the history of prior bowel resection was lower in the EH group (26.3% [n = 10]) compared with remaining groups (TH: 45.8%; RH: 43.5%; NH: 40.9%), but across the groups both of these differences were not statistically significant (*P* = .27 and *P* = .29, respectively).

### Long-term risk of surgery

The cumulative probabilities of surgery were statistically different across time for the 4 healing categories, with probabilities lowest for patients with TH at 0%, 2.3%, and 7.0% at 1, 3 and 5 years, respectively, and highest for patients with NH (13%, 20.5%, and 26.9% at 1, 3, and 5 years respectively) (*P* = .03). Pairwise comparisons indicate a statistical difference in the probability of surgery across the study duration for TH patients vs NH patients (*P* = .03) and vs EH patients (*P* = .04), but no differences were found among other healing categories (*P* > .05) (Table 3).

**Table 3:** Probabilities of CD-related hospitalization and CD-related surgery at 1, 3, and 5 years based on types of healing.

	1 y	3 y	5 y
**Risk of CD-related hospitalization (%)**			
**No healing**	11.4 (3.7-18.6)	18.9 (9.1-27.6)	23.5 (12.7-32.9)
**Endoscopic healing**	10.5 (0.2-19.8)	15.8 (3.4-26.6)	21.4 (7.1-33.5)
**Radiologic healing**	18.3 (0.3-33.0)	23.4 (3.0-39.4)	29.3 (6.1-46.7)
**Transmural healing**	4.3 (0.0-10.0)	8.8 (0.2-16.7)	13.6 (2.8-23.2)
**Risk of CD-related surgery (%)**			
**No healing**	13.0 (4.7-20.5)	20.5 (10.3-29.6)	26.9 (15.4-36.9)
**Endoscopic healing**	13.2 (1.7-23.3)	18.4 (5.1-29.9)	21.2 (7.0-33.3)
**Radiologic healing**	10.0 (0.0-22.4)	10.0 (0.0-22.4)	17.0 (0.0-33.0)
**Transmural healing**	0.0 (0.0-0.0)	2.3 (0.0-6.6)	7.0 (0.0-5.3)

Values are % (95% confidence interval).

Abbreviation: CD, Crohn’s disease.

On comparison of different types of healing, those who achieved TH had significantly lower risk of requiring CD-related surgery compared, with individuals unable to achieve any form of healing (hazard ratio [HR], 0.01; 95% confidence interval [CI], 0.00-0.35; *P* < .01), but no differences were found among the EH, RH, and NH groups (*P* > .05 for other healing group comparisons) (Table 4 and Figure 1). These findings were consistent when examining risk of surgery using modified Poisson regression, with the relative risk (RR) of requiring surgery by end of study follow-up lower in those achieving TH compared with no healing (RR, 0.89; 95% CI, 0.80-1.00; *P* = .05) but no differences among other groups (Table S1 presents the full results for the modified Poisson regression models).

**Figure 1:**
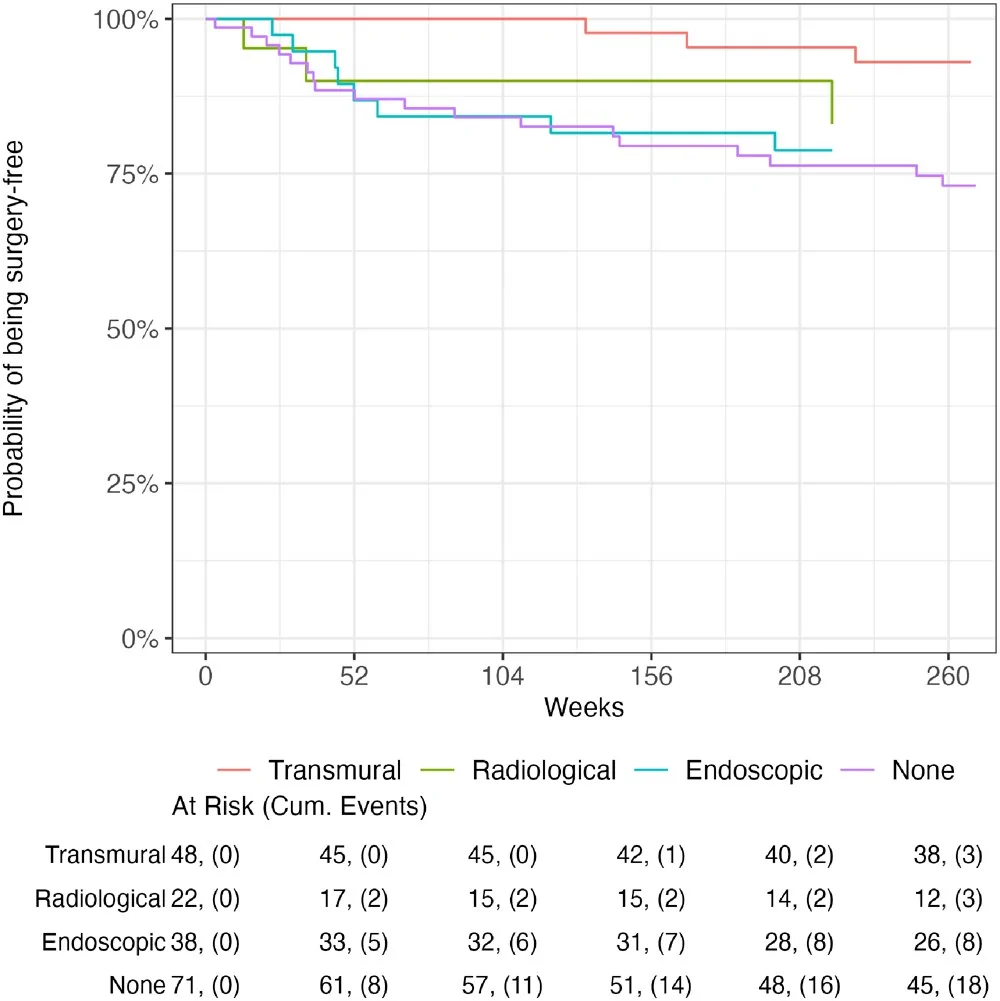
Kaplan-Meier plot showing time to Crohn’s disease–related surgery in patients with different types of healing.

**Table 4:** Cox proportional hazards regression showing risk factors associated with time to surgery.

	Unadjusted		Adjusted	
	HR (95% CI)	*P* value	HR (95% CI)	*P* value
**Type of healing**				
**None**	—	—	—	—
**Endoscopic**	0.91 (0.44-1.89)	.80	1.02 (0.26-4.00)	.98
**Radiologic**	0.50 (0.15-1.66)	.26	0.61 (0.13-2.82)	.53
**Transmural**	**0.24 (0.08-0.71)**	**.01^a^**	**0.01 (0.00-0.35)**	**.009^a^**
**Hb**	—	—	0.99 (0.96-1.03)	.72
**Albumin**	—	—	**0.85 (0.72-0.99)**	**.04^a^**
**CRP >5 mg/L**	—	—	0.47 (0.16-1.36)	.17
**Prior biologic**	—	—	**3.00 (1.10-8.17)**	**.03^a^**
**On biologic**	—	—	0.66 (0.23-1.91)	.44
**Perianal surgery**	—	—	**5.76 (1.08-30.6)**	**.04^a^**
**Inflammation proximal to TI**	—	—	2.97 (0.92-9.65)	.07
**Strictures on endoscopy**	—	—	1.64 (0.54-5.01)	.38
**Current smoker**	—	—	0.54 (0.11-2.64)	.45

Abbreviations: CI, confidence interval; CRP, C-reactive protein; Hb, hemoglobin; HR, hazard ratio; TI, terminal ileum.

a Statistically significant.

Among the factors assessed for their interaction with risk of surgery, increase in albumin at baseline was associated with lower risk of surgery, whereas prior biologic use and history of perianal surgery were associated with increased risk of surgery.

### Long-term risk of hospitalization

The cumulative probability of hospitalization because of a CD flare was lowest for patients with TH at 1, 3, and 5 years, at 4.3%, 8.8%, and 13.6%, respectively (Table 3). However, there was no statistically significant difference in the cumulative probability of hospitalization among healing categories over time (*P* = .67) or in the risk of CD-related hospitalization among different groups (Table 5 and Figure 2). Findings were consistent across both Cox proportional hazards models and modified Poisson regression (Table S2 depicts results from the modified Poisson regression).

**Figure 2:**
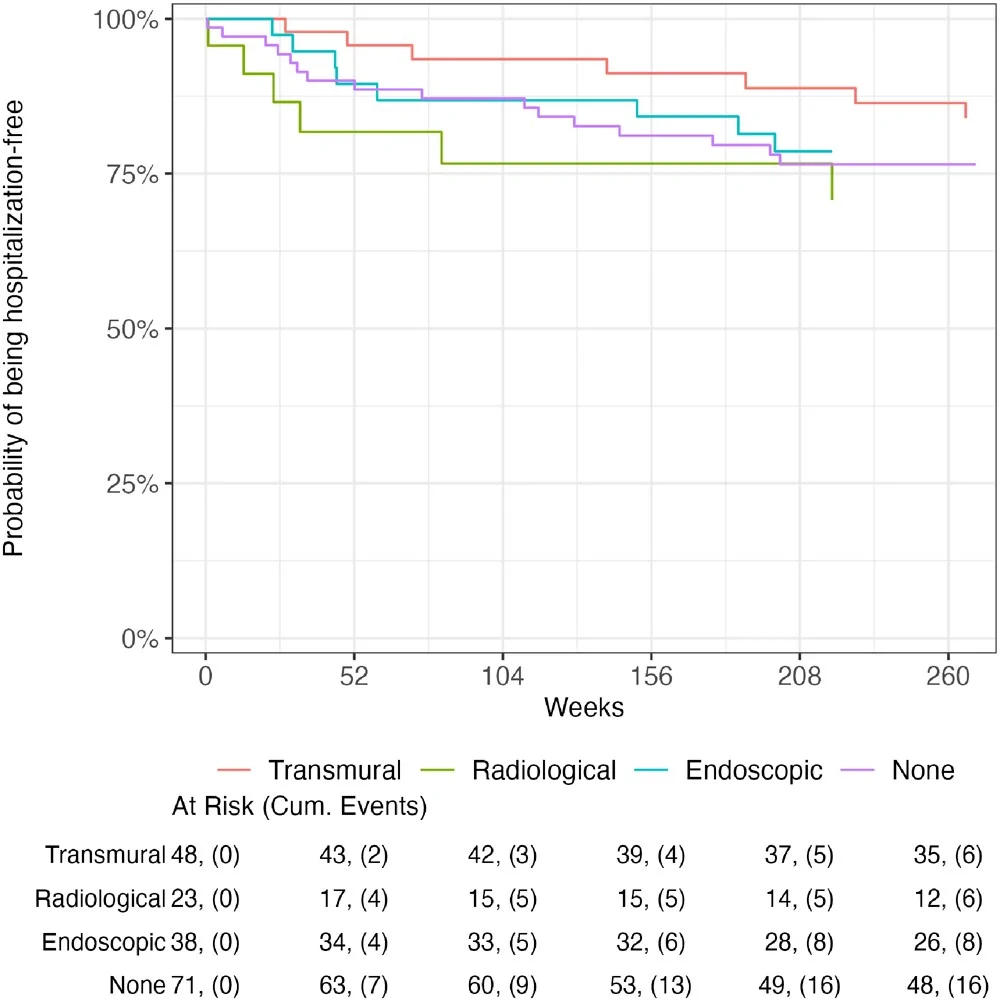
Kaplan-Meier plot showing time to Crohn’s disease–related hospitalization in patients with different types of healing.

**Table 5:** Cox proportional hazards regression showing risk factors associated with time to hospitalization.

	Unadjusted		Adjusted	
	HR (95% CI)	*P* value	HR (95% CI)	*P* value
**Type of healing**				
**No healing**	—	—	—	—
**Endoscopic**	1.39 (0.48-4.01)	.54	4.39 (0.95-20.3)	.06
**Radiologic**	0.89 (0.18-4.41)	.89	1.64 (0.27-10.1)	.59
**Transmural**	0.64 (0.19-2.08)	.454	1.83 (0.36-9.61)	.47
**CRP >5 mg/L**	1.65 (0.60-4.56)	.33	1.83 (0.48-6.94)	.38
**Hb**	—	—	0.98 (0.96-1.01)	.30
**Albumin**	—	—	0.92 (0.81-1.05)	.22
**Prior biologic**	—	—	**3.72 (1.50-9.23)**	**.01^a^**
**On biologic**	—	—	**0.34 (0.13-0.94)**	**.04^a^**
**Perianal surgery**	—	—	**4.73 (1.16-19.2)**	**.03^a^**
**Inflammation proximal to TI**	—	—	1.75 (0.64-4.76)	.27
**Strictures on endoscopy**	—	—	2.25 (0.84-6.07)	.11
**Current smoker**	—	—	0.32 (0.07-1.48)	.15

Abbreviations: CI, confidence interval; CRP, C-reactive protein; Hb, hemoglobin; HR, hazard ratio; TI, terminal ileum.

a Statistically significant.

Among the factors assessed for their interaction for risk of hospitalization, there was statistically significant interaction of prior biologic usage (HR, 3.50; 95% CI, 1.46-8.38) and history of perianal surgery (HR, 4.73; 95% CI, 1.16-19.2) with increased risk of hospitalization (Table 4) whereas current biologic usage was associated with reduced risk (HR, 0.34; 95% CI, 0.13-0.94).

As a sensitivity analysis, we examined the impact of exclusion of type of biologic a patient was using during study follow-up for the analysis. No meaningful difference in estimated models was found from the exclusion of type of biologic. A stratified analysis by type of biologic was also examined and adjusted for potential confounders, and results can be found in Table S3.

## Discussion

In this study, we found that patients with CD who achieved TH, defined as the absence of inflammation on both ileocolonoscopy and cross-sectional imaging, had superior long-term outcomes compared with those with EH or RH alone or NH. The cumulative probabilities of CD-related surgery and hospitalization for patients with TH were 0% and 4.3% at 1 year, respectively. Our findings align with previously published literature, supporting the notion that TH can be considered a potential future treatment target.

The primary goals of medical therapy in patients with CD are the achievement and maintenance of remission, prevention of disease progression, and quality-of-life improvement. The ideal treatment target(s) to achieve these goals remains uncertain. Clinical symptom-based treatment targets are undoubtedly important from patients’ perspective; however, approximately half of patients with CD in clinical remission continue to have endoscopic disease activity, and one-fifth of patients in endoscopic and/or biomarker remission can have active symptoms.^14^ Similarly, patients with EH can continue to have evidence of transmural inflammation on cross-sectional imaging. An observational study by Samuel et al^15^ reported active small bowel CD in 53.7% (n = 36 of 67) despite normal ileocolonoscopy due to skipping of the terminal ileum, or the inflammation was restricted to the intramural part of the ileum normal ileocolonoscopy. In another study, small bowel lesions were detected on cross-sectional imaging beyond the reach of ileocolonoscopy in 22.3% (n = 45 of 202) of cases, indicating that ileocolonoscopy alone is insufficient for complete assessment of bowel inflammation.^8^ Therapeutic strategies aimed at clinical symptom-based targets alone have failed to modify the course of CD when considering avoidance of surgery as a surrogate treatment endpoint.^16^ Consequently, expert consensus recommends targeting EH. However, accumulating evidence indicates that achieving TH may be associated with better long-term outcomes than EH and may even change the natural course of the disease by decreasing the need for surgery and reducing hospitalization rates in CD.^9^ In a study by Fernandes et al^17^ that included 404 patients with CD, 20.8% were classified as having transmural remission, 23.3% were classified as having isolated endoscopic remission, 13.6% were classified as having isolated radiologic remission, and the remaining 42.3% were classified as having no remission. In this study, transmural remission defined by inactive colonoscopy and MRE was statistically superior to isolated endoscopic remission (inactive endoscopy with active MRE) in hospitalization, surgery, need for steroids, and treatment escalation at 5 years.^17^ Moreover, transmural remission consistently showed superior outcomes compared with no remission. Similarly, in the study by Lafeuille et al,^11^ TH (HR, 0.05; 95% CI, 0.00-0.40; *P* = .002) and MRI healing (HR, 0.09; 95% CI, 0.00-0.47; *P* = .005) were associated with lower risk of bowel damage progression than EH. In addition, achieving TH or MRI healing reduced the risk of major outcomes compared with EH, and decreased risk of relapse-related drug discontinuation compared with those with EH. These findings are consistent with the results of our study. Similar findings have also been reported in pediatric CD studies.^18^

Although TH is increasingly recognized and advocated as a potential future treatment target, considerable heterogeneity remains regarding the optimal assessment tool and its definition. MRE is generally regarded as the gold standard for assessing intestinal inflammation, offering advantages such as lack of radiation exposure, superior soft tissue contrast, and functional motility assessment, although CTE is also widely used modality for evaluation of bowel inflammation; however, it is associated with radiation exposure. Recently, IUS has been shown to be sensitive and specific for assessing inflammatory disease activity in IBD. In the METRIC study (Diagnostic Accuracy of Magnetic Resonance Enterography and Small Bowel Ultrasound for the Extent and Activity of Newly Diagnosed and Relapsed Crohn’s Disease), IUS has demonstrated similar sensitivity and specificity for detecting small bowel and colonic CD compared with MRE.^19^ However, MRE outperformed IUS for evaluation of CD in small bowel inflammation. IUS offers several advantages, including real-time assessment, suitability as a point-of-care tool in clinics, cost-effectiveness, and patient friendliness. In the recent systematic review by Geyl et al,^9^ out of 17 studies that reported TH, only seven studies used the IUS definition. However, there is no universally accepted definition of TH. The most commonly used imaging parameter to define TH is bowel wall thickness (≤3 mm). Some studies have used a more stringent definition of absence of any sign of inflammation including complications, and even incorporated absence of inflammation on ileocolonoscopy. A recent international MRI consensus highlighted heterogeneity of TH definition in the literature and recommended 4 definitions of TH in clinical trials based on bowel wall thickness and additional features on MRI.^20^ Recently, the DEVISE-CD project developed the modified Clermont score (C-score) based on bowel thickness, enlarged lymph nodes, and presence of ulcers.^21^ TH and transmural response according to C-score were predictive of favorable long-term outcomes. Ongoing studies, such as TRENCH1 (NCT05903066), a multicenter prospective cross-sectional study in patients with CD aiming to develop an objective and reproducible system for evaluating TH, are expected to help standardize its definition.

Recent advancements, including the availability of multiple classes of advanced therapies and the development of new agents, have enabled treat-to-target management strategies through timely switching to alternative treatments. Despite this, achieving TH is more challenging than clinical improvement or EH. In a retrospective observational study, a fewer number of patients could achieve TH (21%) compared with clinical remission (73%) and endoscopic remission (48%).^22^ In real-world studies, TH varies from 20% to 40%.^23^ Therefore, future studies should evaluate treatment strategies aimed at increasing the likelihood of achieving TH. Emerging evidence suggests that treating early in the disease course increases the chances of attaining TH. In a recent multicentric retrospective observational study, authors demonstrated that early biologic therapy within 12 months was associated with a higher probability of TH (32% vs 14%).^22^ Similar results were reported in another propensity-matched analysis in which early top-down therapy was shown to be associated with higher rates of transmural remission (33.3% vs 18.1%).^24^ An ongoing randomized controlled trial designed to evaluate treatment to target TH in patients with CD using IUS (VECTORS [NCT06257706]) is expected to provide valuable insights on this.

Effectiveness of various classes of advanced therapies in achieving TH also needs to be investigated. In the existing literature, the majority of studies report limited achievement of TH with anti-tumor necrosis factor therapies and different classes of advanced therapies. In a prospective multicenter study from Italy, TH at 12 months after adalimumab was 26.8% and was 37% after infliximab, 27.2% after vedolizumab, and 20% after ustekinumab.^25^ These limited data indicate that comparative efficacy of different advanced therapies in achieving TH needs to be evaluated in future studies.

We acknowledge several limitations of our study, including its retrospective design and the inherent selection bias from recruiting patients at a tertiary academic center. Follow-up cross-sectional imaging was not available to evaluate progression of bowel damage or complications. Furthermore, our stringent definition of TH may have led to underestimation, as the absence of complications such as fistulas or strictures was included in the criteria. As fistulas and strictures may persist despite effective medical therapy, such patients would not have fulfilled the criteria for TH. The time interval between colonoscopy and imaging may have contributed to discrepancies in disease activity for some participants due to progression or improvement of CD. However, as the median interval was only 1 month, it is unlikely to have significantly impacted the assessment of healing. Last, we did not stratify outcomes based on disease phenotype. Despite these limitations, our study makes a meaningful contribution to the limited body of literature by strengthening the evidence on TH, highlighting its association with long-term outcomes and underscoring the need for standardized definitions and validated assessment tools.

In conclusion, TH is associated with substantially better long-term outcomes compared with healing assessed solely by ileocolonoscopy or cross-sectional imaging. It is increasingly recognized as a potential therapeutic target in CD but requires further evaluation in well-designed prospective studies. The considerable heterogeneity in current definitions limits comparability across studies, underscoring the need for a uniform consensus on its assessment. Moreover, future research should investigate the relative efficacy of advanced therapies in achieving TH to better guide clinical decision making.

## Supplementary Material

izag114_Supplementary_Data

## Data Availability

The data that support the findings of this study are available from the corresponding author upon reasonable request.
