# Supplementary material for: Patients with Crohn’s disease achieving transmural healing experience superior long-term outcomes compared with those with endoscopic or radiologic healing alone
Source: Inflamm Bowel Dis. 2026 Jul 3;32(11):2160–8. doi: 10.1093/ibd/izag114 (PMC13722116; doi:10.1093/ibd/izag114)
Supplement: Supplementary file 1 [file izag114_supplementary_data.docx]

**Supplementary Figure 1**

**Supplementary Table 1.** Modified Poisson regression showing relative risk of surgery

|  | Unadjusted | | Adjusted | |
| --- | --- | --- | --- | --- |
|  | **RR (95% CI)** | **p-value** | **RR (95% CI)** | **P-value** |
| Type of Healing |  |  |  |  |
| None | - | - | - | - |
| Endoscopic | 1.00 (0.87–1.14) | 0.95 | 1.03 (0.83—1.26) | 0.81 |
| Radiological | 0.88 (0.75–1.02) | 0.09 | 0.93 (0.77—1.13) | 0.48 |
| Transmural | **0.84 (0.75–0.93)** | **0.001** | **0.89 (0.80—1.00)** | **0.05** |
| Hb |  |  | 1.00 (1.00—1.00) | 0.97 |
| Albumin |  |  | 0.98 (0.97—1.00) | 0.03 |
| Prior Biologic |  |  | 1.11 (0.95—1.29) | 0.18 |
| On Biologic at Study Start |  |  | 0.95 (0.84—1.07) | 0.39 |
| Inflammation Proximal to T1 |  |  | 1.10 (0.93—1.30) | 0.28 |
| Strictures on Endoscopy |  |  | 1.09 (0.93—1.28) | 0.30 |
| Current Smoker |  |  | 0.91 (0.78—1.05) | 0.18 |

**Supplementary Table 2.** Modified Poisson regression showing relative risk of hospitalization

|  | Unadjusted | | Adjusted | |
| --- | --- | --- | --- | --- |
|  | **RR (95% CI)** | **p-value** | **RR (95% CI)** | **P-value** |
| Type of Healing |  |  |  |  |
| None | - | - | - | - |
| Endoscopic | 1.10 (0.90–1.35) | 0.33 | 1.23 (0.99–1.51) | 0.06 |
| Radiological | 0.95 (0.76–1.20) | 0.69 | 1.07 (0.81–1.42) | 0.64 |
| Transmural | 0.95 (0.80–1.12) | 0.53 | 1.10 (0.93–1.32) | 0.27 |
| CRP >5 | 1.11 (0.92–1.34) | 0.28 | 1.12 (0.90–1.38) | 0.31 |
| Healing x CRP |  |  |  |  |
| Endoscopic x CRP>5 | 0.80 (0.58–2.70) | 0.18 | **0.67. (0.46—0.99)** | **0.05** |
| Radiologic x CRP>5 | 1.03 (0.72–1.47) | 0.87 | 0.97 (0.63–1.51) | 0.90 |
| Transmural x CRP >5 | 0.97 (0.70–1.35) | 0.87 | **0.73 (0.54—0.98)** | **0.04** |
| Hb |  |  | 1.00 (1.00–1.00) | 0.69 |
| Albumin |  |  | 0.98 (0.97–1.00) | 0.07 |
| Prior Biologic |  |  | 1.19 (1.02–1.40) | 0.03 |
| On Biologic at Study Start |  |  | 0.87 (0.76–0.99) | 0.04 |
| Inflammation Proximal to T1 |  |  | 1.06 (0.88–1.28) | 0.58 |
| Strictures on Endoscopy |  |  | 1.12 (0.94–1.33) | 0.19 |
| Current Smoker |  |  | 0.90 (0.76–1.07) | 0.23 |

Supplemental Table 3. Cox proportional hazard regression for the time to surgery or hospitalization for type of healing, stratified by type of biologic.

|  | None (n=33) | Anti-TNF (n=85) | IL-23 (n=43) | Interlukin (n=19) |
| --- | --- | --- | --- | --- |
| Type of Healing | **Number of Surgical Events per Number of Healing Patients** | | | |
| None (n=71) | 2/9 | 7/23 | 9/29 | 3/10 |
| Endoscopic (n=38) | 2/7 | 6/21 | 0/7 | 3/3 |
| Radiological (n=23) | 0/4 | 2/14 | 0/3 | 1/2 |
| Transmural (n=48) | 0/13 | 3/27 | 1/4 | 0/4 |
| Type of Healing | **Time to Surgery** | | | |
| None (n=71) | - | - | - | - |
| Endoscopic (n=38) | - | 0.07 (0.00—5.74) | - | 5.88 (0.96—35.9) |
| Radiological (n=23) | - | 0.00 (0.00—7.28) | - | 3.52 (0.30—41.2) |
| Transmural (n=48) | - | 0.00 (0.00—139.0) | - | 0.00 (0.00-Inf) |
| Type of Healing | **Number of Hospitalization Events per Number of Healing Patients** | | | |
| None (n=71) | 1/9 | 7/23 | 8/21 | 4/10 |
| Endoscopic (n=38) | 2/7 | 6/21 | 0/7 | 3/3 |
| Radiological (n=23) | 0/4 | 4/14 | 0/3 | 2/2 |
| Transmural (n=48) | 0/13 | 7/27 | 1/4 | 1/4 |
| Type of Healing | **Time to Hospitalization** | | | |
| None (n=71) | - | - | - | - |
| Endoscopic (n=38) | - | 0.79 (0.12—5.27) | - | 1.53 (0.28—8.40) |
| Radiological (n=23) | - | 0.56 (0.08—4.20) | - | 5.52 (0.89—34.3) |
| Transmural (n=48) | - | 0.19 (0.03—1.42) | - | 0.49 (0.05—4.39) |

*Estimates presented as hazard rates (HR) and 95% confidence intervals.

*Models for anti-TNF patients are adjusted for confounders

*Due to low numbers of events, models for patients on no biologics or on IL-23 were not estimable; for patients on Interlukins, models were only estimable in unadjusted models.
